# Synergistic growth inhibition by Iressa and Rapamycin is modulated by VHL mutations in renal cell carcinoma

**DOI:** 10.1038/sj.bjc.6602646

**Published:** 2005-06-14

**Authors:** R M Gemmill, M Zhou, L Costa, C Korch, R M Bukowski, H A Drabkin

**Affiliations:** 1 Division of Medical Oncology, University of Colorado at Denver and Health Sciences and Cancer Centers, Mail Stop 8117, PO Box 6511, Aurora, CO 80045-0511, USA; 2 Cleveland Clinic Taussig Cancer Center, Cleveland Clinic Foundation, 9500 Euclid Avenue, Cleveland, OH 44195, USA

**Keywords:** protein biosynthesis, kidney neoplasms, epidermal growth factor receptor, mitogen-activated protein kinases

## Abstract

Epidermal growth factor receptor (EGFR) and tumour growth factor alpha (TGF*α*) are frequently overexpressed in renal cell carcinoma (RCC) yet responses to single-agent EGFR inhibitors are uncommon. Although von Hippel–Lindau (VHL) mutations are predominant, RCC also develops in individuals with tuberous sclerosis (TSC). Tuberous sclerosis mutations activate mammalian target of rapamycin (mTOR) and biochemically resemble VHL alterations. We found that RCC cell lines expressed EGFR mRNA in the near-absence of other ErbB family members. Combined EGFR and mTOR inhibition synergistically impaired growth in a VHL-dependent manner. Iressa blocked ERK1/2 phosphorylation specifically in wt-VHL cells, whereas rapamycin inhibited phospho-RPS6 and 4E-BP1 irrespective of VHL. In contrast, phospho-AKT was resistant to these agents and MYC translation initiation (polysome binding) was similarly unaffected unless AKT was inhibited. Primary RCCs *vs* cell lines contained similar amounts of phospho-ERK1/2, much higher levels of ErbB-3, less phospho-AKT, and no evidence of phospho-RPS6, suggesting that mTOR activity was reduced. A subset of tumours and cell lines expressed elevated eIF4E in the absence of upstream activation. Despite similar amounts of EGFR mRNA, cell lines (*vs* tumours) overexpressed EGFR protein. In the paired cell lines, PRC3 and WT8, EGFR protein was elevated post-transcriptionally in the VHL mutant and EGF-stimulated phosphorylation was prolonged. We propose that combined EGFR and mTOR inhibitors may be useful in the subset of RCCs with wt-VHL. However, apparent differences between primary tumours and cell lines require further investigation.

Renal cell carcinoma (RCC) afflicts annually over 30 000 individuals in North America with 12 000 deaths. Approximately 70–75% of RCCs have clear-cell morphology and one-half or more of these have mutations or epigenetic silencing of the von Hippel–Lindau (VHL) gene (Latif *et al*, 1993; Herman *et al*, 1994). As part of an E3 ubiquitin ligase (VBC), VHL targets specific substrates for polyubiquitylation and subsequent proteasome-mediated destruction with hypoxia inducible factor alpha subunits (HIF*α*) being the best characterized (Kaelin, 2003). Deregulation of HIF*α* appears to be essential to the disease process (Kondo *et al*, 2002; Maranchie *et al*, 2002), although other VHL targets including fibronectin (Ohh *et al*, 1998), hnRNP molecules (Pioli and Rigby, 2001) and deubiquitinating enzymes (Li *et al*, 2002) may contribute.

Overexpression of the EGF receptor (EGFR) in RCC has been recognised for some time and EGFR signalling is mitogenic for malignant and normal renal tubular cells (Gomella *et al*, 1989; Humes *et al*, 1991; Uhlman *et al*, 1995; de Paulsen *et al*, 2001). TGF*α*, an EGFR ligand under transcriptional control by HIF, is constitutively expressed in VHL mutant cells (Knebelmann *et al*, 1998; de Paulsen *et al*, 2001; Gunaratnam *et al*, 2003). These observations have led to clinical trials of EGFR inhibitors in RCC (Drucker *et al*, 2003; Motzer *et al*, 2003; Foon *et al*, 2004; Rowinsky *et al*, 2004; Amato, 2005). However, as single –agents, the response rates have been low although stable disease may be prolonged. In contrast, the combination of EGFR and VEGF inhibitors may be more active than either agent alone (Hidalgo, 2003).

Downstream effectors of EGFR signalling include the Ras/Raf/MAP kinase and phosphatidyl inositol 3-OH kinase (PI3K) pathways. PI3K signalling leads to phosphorylation and activation of AKT, which blocks apoptosis (Thompson and Thompson, 2004), promotes cytoplasmic sequestration of FoxO transcription factors (Brunet *et al*, 1999), and activates protein translation initiation via the mammalian target of rapamycin (mTOR) (Hay and Sonenberg, 2004). Phosphatase and tensin homolog (PTEN) mutations, which activate AKT, have been described in RCC in association with more aggressive disease (Kondo *et al*, 2001; Shin Lee *et al*, 2003). Mammalian target of rapamycin (mTOR) is inhibited by the tuberous sclerosis (TSC) complex, which, in turn, is inhibited by AKT and patients with TSC mutations have an increased incidence of RCC (Al-Saleem *et al*, 1998). Biochemically, mouse embryo fibroblasts defective in TSC resemble RCCs with VHL mutations as they express elevated levels of HIF*α*, VEGF and glycolytic proteins (Brugarolas *et al*, 2003). Tuberous sclerosis mutant cells have an exaggerated HIF*α* response to hypoxia, which is blocked by treatment with rapamycin. A rapamycin prodrug, CCI-779 (Wyeth), has been examined as a single agent in RCC (Atkins *et al*, 2004). The response rate was low (7%) but included one complete response. Rapamycin has shown additive or synergistic activity when combined with other tyrosine kinase inhibitors such as Gleevec and PKC412 (Mohi *et al*, 2004). Rapamycin also enhances chemotherapy responses in breast cancer cell lines (Mondesire *et al*, 2004) and in Myc-driven murine lymphomas (Ruggero *et al*, 2004). Tumours with loss of PTEN, loss of p53 or amplification of GLI may be particularly sensitive to rapamycin (Hosoi *et al*, 1999; Louro *et al*, 1999; Neshat *et al*, 2001).

Mammalian target of rapamycin, a serine/threonine kinase, positively affects protein translation initiation by phosphorylating two key regulators, 4E-BP1 and p70S6 kinase (S6K1) (Hay and Sonenberg, 2004). eIF4E binds the 5′-methyl cap (^me7^GpppN) modification of mRNAs and is essential for cap-dependent translation initiation. Unphosphorylated 4E-BP1 binds and inhibits eIF4E. Overexpression of eIF4E can transform NIH 3T3 cells (Lazaris-Karatzas *et al*, 1990) and can cause rapamycin-sensitive murine lymphomas to become rapamycin-resistant (Ruggero *et al*, 2004). In contrast to inhibiting 4E-BP1 function, S6K is activated by mTOR which leads to the phosphorylation of ribosomal protein S6 (RPS6) and increased translation of mRNAs, particularly those encoding components of the translation machinery itself (e.g. RPS19). Other signalling pathways also affect protein translation initiation. For instance, ERK1/2 activation affects multiple translation initiation components including S6K, p90 RSK, MNK and 4E-BP1, among others (Roux and Blenis, 2004). Also, in addition to eIF4E-mediated cap-dependent initiation, there are internal ribosome entry sites present in several mRNAs important in RCC such as MYC, VEGF and Cyclin D1 (Stoneley and Willis, 2004; Shi *et al*, 2005), which may allow translation initiation in a cap-independent manner.

We hypothesised that the combination of an EGFR inhibitor plus rapamycin might inhibit growth in RCC cell lines in a synergistic manner. This proved to be correct but synergy at low drug concentrations was only observed in cell lines with wild-type (wt) VHL. Mechanistically, loss of ERK1/2 and RPS6 phosphorylation following EGFR inhibition was confined to wt-VHL cells. Rapamycin more effectively inhibited phospho-4E-BP1 and this was VHL-independent. In contrast, neither agent inhibited phospho-AKT and similarly, neither agent nor combination blocked MYC translation initiation. However, blocking AKT with the PI3K inhibitor, LY-294002, substantially downregulated the association of MYC mRNA with ribosomes (i.e. polysome loading). Among seven parental RCC cell lines analysed biochemically, we found one with overexpression of eIF4E. With these results in mind, we examined a series of patient tumours. Extracellular signal-regulated kinase (ERK) and AKT pathways showed similar evidence for activation, although levels of phospho-ERK were relatively much greater. One tumour contained elevated eIF4E and several biochemical characteristics closely matched the one cell line with eIF4E overexpression. Most tumours, as expected, contained elevated EGFR protein compared to normal kidney, although these levels were substantially lower than in RCC cell lines. Interestingly, phospho-RPS6 was markedly reduced in tumours, suggesting that mTOR activity was lower than observed in cell lines. These preclinical findings in cell lines suggest that combined EGFR and mTOR inhibitors may benefit a particular subset of RCC patients but the lack of AKT inhibition potentially limits the response. Apparent differences between primary tumours and cell lines suggest that it would be particularly useful to examine tumours pre- and post-treatment with phospho-specific antibodies to ERK, AKT and RPS6 (or S6K).

## METHODS

### Cell lines, genotyping and patient tumours

786-O cells were obtained from the American Type Culture Collection (Manassas, VA, USA). Stable 786-O transfectants, WT8 (containing wt-VHL) and PRC3 (vector control), were kindly provided by Dr William Kaelin. Additional wt-VHL transfectants, MPR6 and MEA2, were kindly provided by Dr Robert D Burk. 786-O cells were grown in Dulbecco's modified Eagle's medium (DMEM) plus 10% fetal calf serum (FCS). WT8, PRC3, MPR6 and MEA2 were maintained in the same medium plus 1000 *μ*g ml^−1^ G418. SKRC-02, SKRC-17, SKRC-39 and SKRC-45 were kindly provided by Dr Elisabeth Stockert (Tumour Cell Bank, Memorial Sloan-Kettering Cancer Center, New York, NY, USA) and maintained in RPMI-1640 with 10% FCS. ACHN was grown in McCoy's medium with 15% FCS and KRCY was grown in RPMI-1640 with 15% FCS. Cells were cultured under standard conditions in a humidified 5% CO_2_ atmosphere. Rapamycin and LY294002 were obtained from Sigma, St. Louis, MO, USA. EGF from Chemicon, Int., Temecula, CA, USA, while ZD-1839 was obtained from Astra-Zeneca, Inc., Wilmington, DE, USA, through the auspices of Dr Paul Bunn. Cell lines were confirmed to be of independent origin by either microsatellite genotyping analysis or VHL mutation determination using DNA sequencing. Genotyping analysis was performed by the University of Colorado Cancer Center DNA Sequencing & Analysis Core using the Applied Biosystems (Foster City, CA, USA). AmpF*l*STR Profiler Plus PCR amplification kit (No. 4303326). The microsatellite genotyping analysis was performed as indicated by the manufacturer. The following 10 loci were examined: D3S1358, vWA, FGA, Amelogenin, D8S1179, D21S11, D18S551, D5S818, D13S317 and D7S820. If a second allele (or Y chromosome in the case of Amelogenin) was not detected, it is listed as ‘nd’. The results (i.e. allele 1/allele 2) for each locus listed above in order are: SKRC2: 17/nd, 15/17, 20/nd, X/nd, 8/nd, 28/30, 12/19, 12/nd, 11/nd, 9/11; SKRC12: 15/nd, 14/16, 20/22, X/nd, 13/14, 29/nd, 12/16, 11/12, 11/13, 8/9; SKRC17: 15/nd, 14/16, 22/nd, X/nd, 13/14, 30/32.2, 15/18, 11/12, 11/12, 8/12; SKRC39: 16/nd, 17/nd, 24/nd, X/nd, 13/nd, 29/31.2, 14/15, 13/nd, 11/nd, 8/11; SKRC45: 15/nd, 15/16, 22/23, X/nd, 12/14, 27/28, 15/nd, 12/nd, 11/12, 12/nd; ACHN: 17/nd, 16/17, 22/nd, X/nd, 12/nd, 30/nd, 16/nd, 12/nd, 12/nd, 9/11; A498: 15/nd, 18/nd, 18/20, X/nd, 13/15, 28/32, 17/nd, 11/nd, 12/nd, 10/11; CAKI2: 14/nd, 16/17, 22/nd, X/Y, 10/nd, 27/31, 17/nd, 11/nd, 10/nd, 12/nd; KRCY: 15/nd, 17/18, 22/nd, X/nd, 14/15, 29/30.2, 14/nd, 12/13, 12/nd, 8/12; KV6: 14/nd, 16/nd, 21/22, X/nd, 12/15, 29/nd, 16/17, 12/nd, 9/13, 9/10.

Frozen sections from 12 RCC tumours and adjacent normal-appearing kidney were processed for Western blot, genomic DNA isolation and quantitative RT–PCR by pulverisation in liquid N_2_ and storage in aliquots at −80°C. Pathological parameters for this tumour cohort are provided in Table 1.

### MTT assays

Microtitre plates were seeded with 2000 cells per well on day 1. Agents and vehicle were added on day 2 and growth continued for an additional 5 days. MTT reagent (3- (4,5-Dimethylthiazol-2-yl)-2,5-diphenyl-tetrazolium bromide, Sigma) was then added to 400 *μ*g ml^−1^ and incorporated for 2–4 h. Deposited dye was solubilised with 75% isopropanol, 240 mM HCl and the absorbance measured at 490 nm in a 96-well plate reader (Molecular Devices, Inc., Sunnyvale, CA, USA). The combination index was calculated by CALCUSYN (Chou and Talalay, 1984).

### Primers for real-time RT–PCR and VHL sequencing

RNA isolation, cDNA synthesis and quantitative RT–PCR assays were performed as previously described (Drabkin *et al*, 2002). Primer sequences (5′–3′) were: EGFR (for) AGATGGAGGAAGACGGCGTC, (rev) GGAGTCACCCCTAAATGCCAC; ErbB-2 (for) ACCTACCTGCCCACCAATGC, (rev) GGTGGTATTGTTCAGCGGGTC; ErbB-3 (for) TCCGCTTGACTCAGCTCACC, (rev) CCCTTGCAAACCTCATGACAG; ErbB-4 (for) CCAGCCCAGCGATTCTCAG, (rev) AAGGAGAGGTCCCGGTTGTG; RPS19 (for) CCTCAAAAAGTCCGGGAAGC, (rev) TGGAAGCAGCTCGCGTGTAG; *β*-actin (for) ACCGCGAGAAGATGACCCAG, (rev) AGGTCCAGACGCAGGATGG; MYC (for), ACACCCTTCTCCCTTCGGG, (rev) AGCCGCTCCACATACAGTCC; VEGF (for) CAAGACAAGAAAATCCCTGTGG, (rev) CCTCGGCTTGTCACATCTG; GAPDH (for) TGCACCACCAACTGCTTAGC, (rev) GGCATGGACTGTGGTCATGAG; VHL (for) GGACACACGATGGGCTTCTG, (rev) CAACCTGGAGGCATCGCTC.

Primers used for genomic amplification of VHL were: either PF1 (upstream of promoter): ACTTTATAAGCGTGATGATTGGGTG or Exon 1 (for): CGAAGACTACGGAGGTCGACTC, (rev) CGTGCTATCGTCCCTGCT; Exon 2 (for) GGATTTAGAGCTTTAAGTACGCGCTC, (rev) TGGATACCGTGCCTGACATCA; Exon 3 (for): GTTGTTGGCAAAGCCTCTTGTTC, (rev) CCATCAAAAGCTGAGATGAAAC. Amplification products were treated with ExoSAP-IT (USB Corporation, Cleveland, OH, USA, 1 *μ*l/10 *μ*l PCR reaction for 30 min at37°C followed by 45 min at 80°C) to remove excess primers and dNTPs. The products were sequenced using ABI Prism BigDye terminators version 1.1 on an ABI 3730 automated DNA sequencer. Sequencing of VHL Exon 2 utilised the primer CAGGACGGTCTTGATCTCCTG together with VHL Exon 2 (rev).

### Western blots

Protein lysates were prepared in ice-cold buffer containing 25 mM Tris-HCl (pH 7.2), 150 mM NaCl, 5 mM DTT, 2 mM MgCl_2_, 0.5% NP-40, 1 mM PMSF, 5 *μ*g ml^−1^ aprotinin, 5 *μ*g ml^−1^ leupeptin, 1 *μ*g ml^−1^ pepstatin A, 1 mM 1,10-phenanthroline, 10 mM *N*-ethylmaleimide, 1 mM activated Na-orthovanadate and 1 mM NaF, clarified in a microcentrifuge (5 min at 10K RPM), and protein concentrations measured by the Bradford assay. Samples were denatured in Laemmli buffer, resolved by SDS–PAGE and transferred to PVDF membranes. Membranes were blocked in PBS/0.1% Tween/10% nonfat dry milk (NFDM) and incubated with primary antibodies for 1 h to overnight in PBS/0.1% Tween/1% NFDM. Filters were washed, incubated with HRP-conjugated secondary antibodies, then rewashed extensively in PBS/0.1% Tween. Detection used the ECL Lightning Plus reagent from Amersham, Inc., Piscataway, NJ, USA. The following antibodies were obtained from Cell Signalling, Beverly, MA, USA, and used according to the manufacturer's directions; EGFR, ErbB2, phospho-EGFR-Y845, phospho-EGFR-Y1068, phospho-ERK1/2, phospho-AKT, AKT, phospho-RPS6, RPS6, 4EBP1 and eIF4E. Anti-ERK antibody was obtained from Promega, Inc., Madison, WI, USA, while the anti-HA (Y11), ErbB3 and ErbB4 antibodies were from Santa Cruz Biotechnology, Inc., Santa Cruz, CA, USA. The mouse monoclonal 11E12, directed against the VHL C-terminus, was a kind gift from Dr RD Burk.

### Polysome fractionation on sucrose gradients

Cultured cells treated for 2 h with inhibitors or vehicle were incubated for 5 min in medium containing 0.1 mg ml^−1^ cycloheximide (CHX). Adherent cells were washed three times with ice-cold PBS/0.1 mg ml^−1^ CHX, and harvested with trypsin/0.1 mg ml^−1^ CHX. The cell pellet was lysed for 10 min on ice in 500 *μ*l of 0.3 M NaCl/15 mM Tris-HCl pH 7.5/15 mM MgCl_2_/1% Triton X-100/0.1 mg ml^−1^ CHX and 1 mg ml^−1^ heparin. Following centrifugation (10K, 5 min at 4°C), the clarified supernatant was layered onto a 10–50% sucrose step-gradient prepared the night before containing 300 mM NaCl, 15 mM Tris-HCl, pH 7.5, 15 mM MgCl_2_, 1 mg ml^−1^ heparin and 0.1 mg ml^−1^ CHX. Gradients were centrifuged at 37K RPM for 2.5 h in an SW41 rotor, collected from the top using an ISCO tube piercing device (ISCO, Inc., Lincoln, NE, USA) and fractions monitored by absorbance at 280 nm. RNA was recovered using sequential guanidine-HCl extraction and ethanol precipitation followed by further purification on RNeasy columns (Qiagen, Inc., Alameda, CA, USA) and on-column digestion with RNase-free DNase using conditions recommended by the manufacturer.

## RESULTS

### Epidermal growth factor receptor expression and synergistic growth inhibition by Iressa plus rapamycin

**Figure 1:**
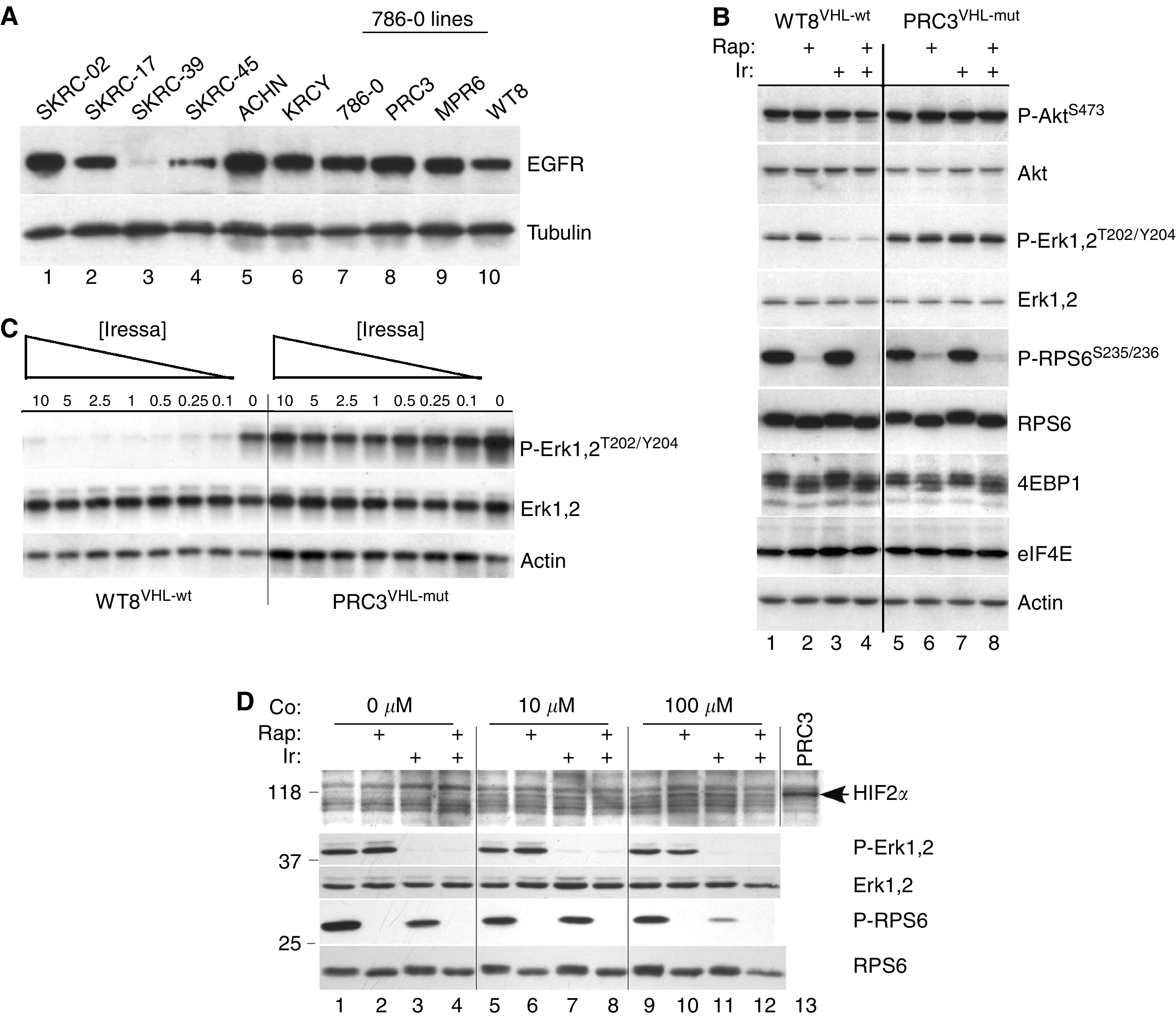
Differential EGFR levels and ERK phosphorylation in RCC cells. (**A**) Aliquots (10 *μ*g) of cell lysates were analysed by Western blot for levels of EGFR protein. PRC3, MPR6 and WT8 are derived from 786-O cells by transfection of empty vector (PRC3) or independent wild-type VHL expression constructs (MPR6 and WT8). Tubulin served as a loading control. (**B**) RCC cell lines WT8^VHL-wt^ and PRC3^VHL-mut^ were grown until 50% confluent, then treated for 2 h with DMSO (lanes 1 and 5), rapamycin (10 nM; lanes 2 and 6), Iressa (10 *μ*M; lanes 3 and 7) or both (lanes 4 and 8). Aliquots were analysed for the indicated total and phospho-proteins. (**C**) WT8^VHL-wt^ and PRC3^VHL-mut^ cells at 50% confluency were treated for 2 h with decreasing doses of Iressa (10–0.1 *μ*M) and analysed as above. (**D**) WT8^VHL-wt^ cells at 50% confluency were treated for 20 h with 10 or 100 *μ*M CoCl_2_, or not, as indicated (Co:). For the last 2 h, selected cultures were treated with rapamycin (10 nM), Iressa (10 *μ*M) or both; control cells were treated with DMSO. Cells were washed twice with PBS, harvested and analysed with the indicated antibodies. One lane of PRC3^VHL-mut^ lysate was included as a positive control for the HIF2*α* antibody (arrow). This lane was not analysed with the other four antibodies.

Previous reports have shown that EGFR expression is elevated in RCC although there is less information concerning other ErbB family members. To address this question, we used real-time RT–PCR to measure EGFR, ErbB-2, ErbB-3 and ErbB-4 mRNA levels in 14 RCC cell lines (Table 2), genetically verified to be of independent origin (see Methods). Expression was normalised to the housekeeping gene, GAPDH, although equivalent results were obtained using *β*-actin (not shown). The results demonstrate that EGFR is the predominantly expressed ErbB family member. Although ErbB-3 was next most highly and frequently expressed, these levels were low in comparison to EGFR. In addition, we determined the VHL mutant status for most of the cell lines and the level of expression for lines with wt-VHL (Table 2). For comparison, VHL mRNA levels were 6.7% of GAPDH in the immortalised renal epithelial cell line, HEK293 (not shown). It is apparent that most lines have either mutant VHL or express this gene at low levels making any potential correlations between VHL and EGFR problematic. Corresponding EGFR protein levels for some of these cell lines are shown in Figure 1A. There was no consistent relationship between EGFR mRNA and protein levels (e.g. SKRC-45 and KRCY). These results suggest that there must be post-transcriptional differences affecting EGFR in RCC. The other three ErbB family members were not detectable by Western analysis.

**Figure 2:**
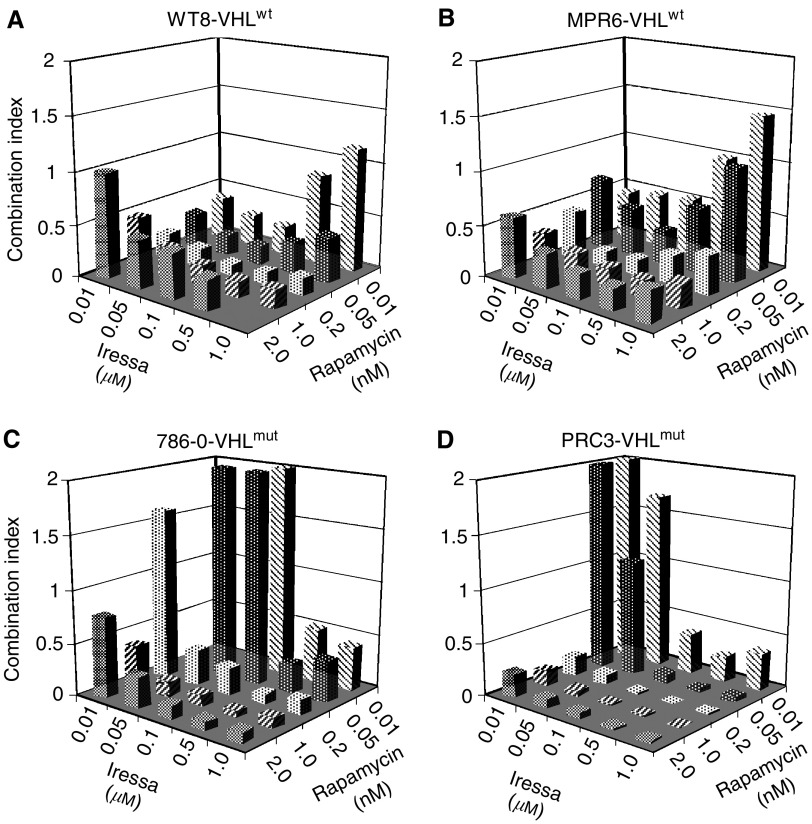
Growth inhibition by Iressa and rapamycin in derivatives of 786-O cells. The parental RCC line 786-O and three stably transfected derivatives were tested for the effects of varying doses of Iressa and rapamycin on growth using MTT assays. MTT absorbance values from single and combined agents were converted to the combination index (see Methods).

The quantitative EGFR results suggested that a selective inhibitor, such as Iressa (Gefitinib, ZD1839), might inhibit growth especially when combined with rapamycin. We tested this hypothesis initially using the paired cell lines, WT8 and PRC3. These lines were derived from 786-O, a clear-cell carcinoma that contains a deletion and frameshift of the VHL gene (Iliopoulos *et al*, 1995). WT8 cells contain stably transfected, HA-tagged VHL (p24 form), while PRC3 cells contain the vector-only control. Low-density cultures of WT8 and PRC3 cells were treated with varying concentrations of Iressa, rapamycin or the combination. After 5 days, growth was measured by MTT assays and the results converted to a combination index using CALCUSYN (Chou and Talalay, 1984). Values much less than 1.0 indicate synergy while values much greater than 1.0 are indicative of antagonism. As shown in Figure 2, Iressa plus rapamycin at various doses synergistically inhibited growth of WT8 cells. Nearly identical results were obtained using an independent stable VHL transfectant, MPR6 (Schoenfeld *et al*, 1998). These results include Iressa concentrations within the reported IC_50_ range for EGFR (i.e. 30–50 nM). At high Iressa and low rapamycin concentrations, a combination index close to 1.0 was obtained (indicating no interaction). In contrast, in the VHL-mutant cell lines 786-O and PRC3, low concentrations of Iressa plus rapamycin were antagonistic. Thus, the introduction of a wt-VHL gene into 786-O cells confers sensitivity (synergy) to the combination of EGFR plus mTOR inhibition at low drug concentrations. Of note, the levels of rapamycin used are readily achievable in patients.

### Biochemical correlates of Iressa and rapamycin exposure

To explore the difference in growth inhibition, we examined selected biochemical targets of EGFR and mTOR signalling in the PRC3 and WT8 cell lines. Short-term (2 h) treatment with Iressa inhibited phosphorylation of ERK1/2 in WT8 but not in PRC3 cells (Figure 1B). Iressa had no effect on AKT or 4E-BP1 phosphorylation in either cell line. Rapamycin, on the other hand, inhibited phosphorylation of both RPS6 and 4E-BP1 in a VHL-independent manner. Thus, in these paired cell lines, we identified biochemical changes that correlated with the observed synergistic growth inhibition in the MTT assays. Since higher concentrations of Iressa (10 *μ*M) were used in these short-term studies, we performed a dose–response analysis of ERK1/2 phosphorylation (Figure 1C). Inhibition was seen in WT8 cells at the lowest Iressa dose used (100 nM) while no inhibition was detected in PRC3.

Differential sensitivity to Iressa in WT8 cells could result from the absence of HIF*α*, a consequence of re-expressing wild-type VHL. As an initial assessment of HIF dependence, we treated WT8 cells with cobalt chloride to chemically induce an hypoxic state and thus upregulate HIF. Cobalt-treated cells were then tested for changes in response to Iressa (Figure 1D). Treatment with 10 and 100 *μ*M CoCl_2_ induced similar levels of the 118 kDa HIF2*α*, the only HIF*α* isoform expressed in 786-O cells (Iliopoulos *et al*, 1995). Induction of HIF2*α* had no effect on the ability of Iressa to inhibit Erk phosphorylation (compare lanes 3, 7 and 11), suggesting that under these conditions, differential sensitivity was not HIF dependent.

**Figure 3:**
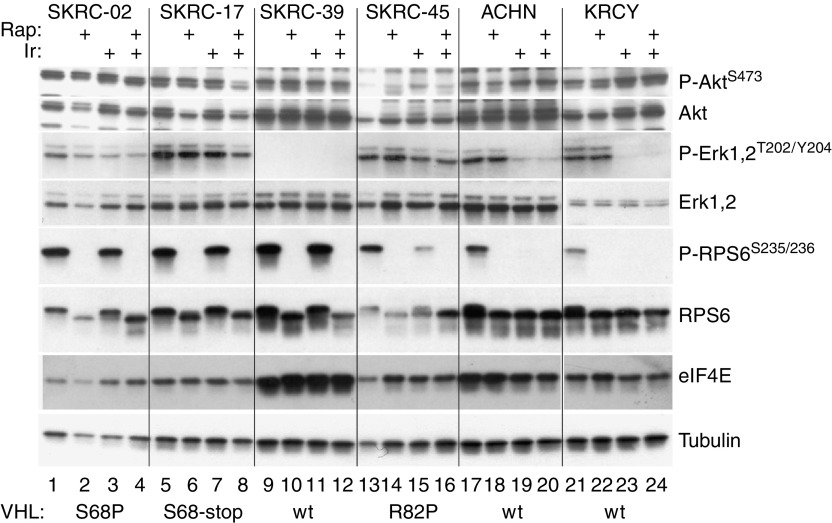
Phospho-protein analysis in additional RCC cell lines. Six RCC cell lines were grown to 50% confluency, then treated for 2 h with DMSO (lanes 1, 5, 9, 13, 17 and 21), rapamycin (10 nM; lanes 2, 6, 10, 14, 18 and 22), Iressa (10 *μ*M; lanes 3, 7, 11, 15, 19 and 23) or both (lanes 4, 8, 12, 16, 20 and 24). Lysates were analysed as in Figure 1. VHL mutational status is indicated along the bottom.

We expanded the biochemical analysis to six additional RCC cell lines (Figure 3). In the wt-VHL lines, ACHN and KRCY, Iressa inhibited both ERK1/2 and RPS6 phosphorylation while phospho-AKT levels were unaffected. In SKRC-39, which expressed the highest level of wt-VHL, the basal protein patterns were strikingly different. These cells overexpressed eIF4E and had low to undetectable levels of phospho-ERK1/2 (Figure 3). Epidermal growth factor receptor levels were also substantially reduced (Figure 1A). Among the three mutant VHL cell lines, only SKRC-45 showed any response to Iressa, consisting of a partial reduction of ERK1/2 and RPS6 phosphorylation. Rapamycin uniformly inhibited phospho-RPS6 regardless of the VHL status. In summary, Iressa was substantially more effective at inhibiting ERK and RPS6 phosphorylation in RCC cell lines with wt-VHL. Using a Wilcoxon Rank Sum Test, this approached but did not reach statistical significance (*P*=0.08) because of the limited number of cell lines.

### EGFR protein levels, phosphorylation and polysome loading of mRNA

**Figure 4:**
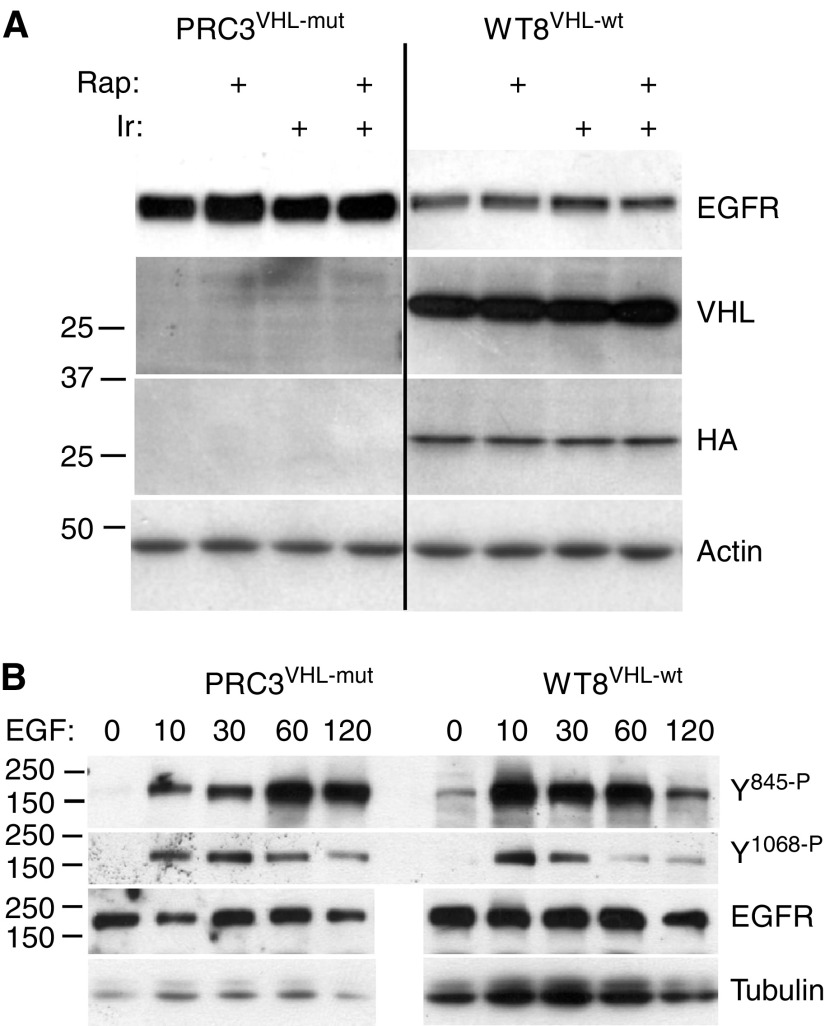
Total and phospho-EGFR levels in PRC3 and WT8. (**A**) Protein lysates, as described in Figure 1, were analysed for total EGFR protein. The blot was stripped and reprobed with anti-VHL and anti-HA antibodies. (**B**) PRC3 and WT8 cultures were grown to 50% confluency, washed with PBS, then starved in 0.1% serum for 2 h. Cells were harvested at the indicated time points (min) after the addition of 10% serum and 50 ng ml^−1^ EGF. More total protein was loaded in the WT8 lanes to compensate for the lower amounts of EGFR in this cell line. Otherwise, equal protein aliquots were analysed for the phosphorylation levels of tyrosine 845 and 1068 using phospho-specific antibodies.

Based on the differential responses to EGFR blockade, we examined levels of EGFR protein in PRC3 and WT8 cells following treatment with Iressa or rapamycin (Figure 4A). Epidermal growth factor receptor levels were higher in the VHL mutant, PRC3, although no changes were induced by treatment. These differences were more apparent at shorter exposures (compare Figures 1A, 2A, 3 and 4A). To examine the phosphorylation dynamics of EGFR, we serum-starved WT8 and PRC3 cells for 2 h, then added back serum plus EGF. Protein lysates were examined at multiple time points using phospho-specific EGFR antibodies (Figure 4B). In these experiments, more protein was loaded for WT8 to compensate for the lower levels of total EGFR. For tyrosine 845, maximum phosphorylation was seen at 10 min in WT8 cells compared to 60 min in PRC3. For tyrosine 1068, the onset of maximum phosphorylation was similar. For both sites, the duration of phosphorylation was prolonged in PRC3. Similar results were obtained in an independent experiment carried out to 180 min after EGF addition with evidence of persistent phosphorylation in PRC3 (not shown). Despite these differences, prior exposure of both cell lines to Iressa for 15 min blocked tyrosine phosphorylation (not shown). Thus, while the presence of wt-VHL in these paired cell lines appears to affect EGFR protein levels and activation, the results do not explain the differential sensitivity to Iressa. One likely possibility is that VHL mutations upregulate signalling from other kinases.

**Figure 5:**
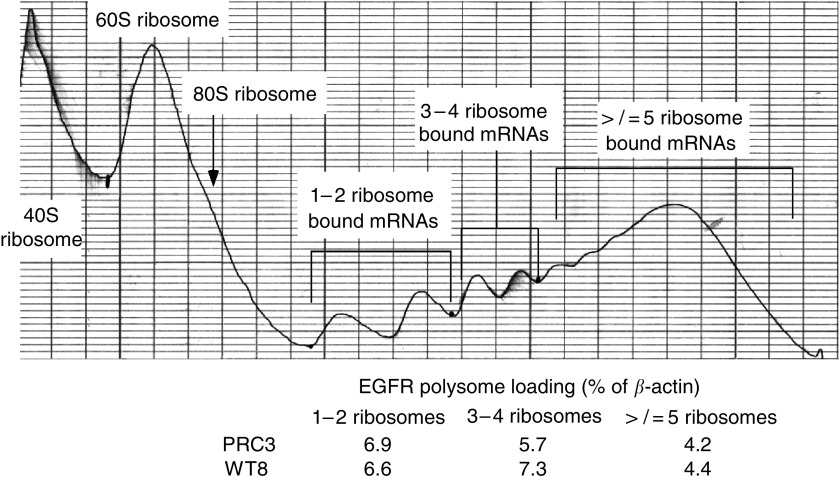
Polysome analysis of WT8 cells. WT8 cells were grown to 70% confluency, then treated for 2 h with DMSO, rapamycin (10 nM), Iressa (10 *μ*M) or both, and harvested for polysome fractionation. A representative A_280_ fractionation profile is shown along with the fractions pooled and the results from quantitative RT–PCR analysis.

To determine whether the elevated EGFR protein in PRC3 was the result of mRNA differences, possibly due to clonal variation, we examined message levels by real-time RT–PCR in parental 786-O, PRC3 and WT8 cells. The analyses, performed in triplicate, demonstrated that there were no differences among the cell lines (Table 3). Like the original series of RCCs examined, ErbB-2, ErbB-3 and ErbB-4 mRNA levels were barely detectable and corresponding proteins were undetectable by Western analysis. In contrast, VEGF expression, as expected, was higher in the VHL mutants 786-O and PRC3 than WT8. These results indicate that the observed differences in EGFR protein levels are post-transcriptional, possibly involving protein translation initiation or degradation. To examine the question of protein translation initiation, we isolated polysome fractions from WT8 and PRC3 cells and analysed these by quantitative real-time RT–PCR (Figure 5). No reproducible differences were identified in ribosome-bound EGFR mRNA between these cell lines. Furthermore, short-term treatment with rapamycin or Iressa had no consistent effects on polysome-associated EGFR message (not shown). These results suggest that the difference in EGFR protein levels (and phosphorylation duration) between PRC3 and WT8 involves internalisation or degradation.

### Downregulation of MYC polysome loading by PI3K inhibition

Activated AKT is known to play an important antiapoptotic role and also positively regulates protein translation initiation. As noted above, EGFR and mTOR inhibitors did not affect phospho-AKT levels and did not have a major effect on EGFR polysome loading. We wished to determine, therefore, whether short-term blockade of AKT (using the PI3K inhibitor LY-294002) would have a demonstrable effect. We chose to focus on MYC translation initiation (polysome loading) in WT8 cells, since this gene is upregulated in RCC and because MYC mRNA contains an internal ribosome entry site, which provides a potential eIF4E-independent means of translation initiation (Shi *et al*, 2005). Also, since WT8 cells were more responsive to Iressa and rapamycin, we combined LY-294002 with these agents. As shown in Table 4, neither Iressa, rapamycin nor the combination substantially reduced MYC mRNA ribosome association and these agents may even have increased polysome loading in some fractions. As a control, we included RPS19 that contains a 5′-untranslated polypyrimidine stretch known to be sensitive to mTOR inhibition (Meyuhas, 2000). RPS19 loading was substantially reduced in all ribosome fractions by both agents. In contrast, MYC polysome loading was reduced only by LY-294002. This effect appeared to be somewhat greater when LY-294002 was combined with rapamycin or rapamycin plus Iressa.

### Analysis of primary RCCs

**Figure 6:**
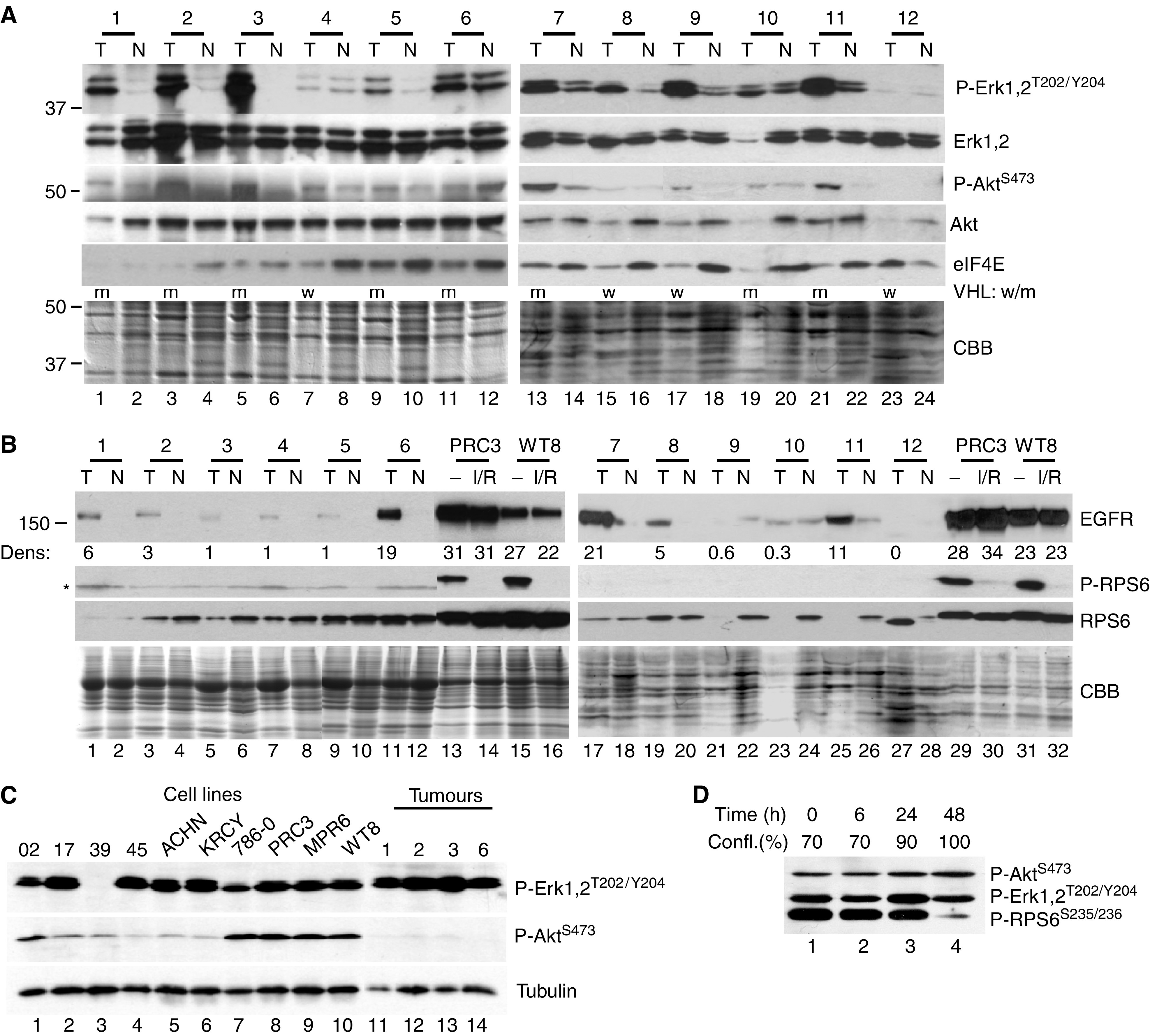
Analysis of primary tumours. (**A**) Protein (10 *μ*g) from 12 matched pairs of renal tumours (T) and normal kidney (N) were analysed with the indicated antibodies on two separate sets of blots. Equivalent gels were stained with Coomassie blue (CBB) for loading. The low protein content of tumour #10 indicates that relative Erk phosphorylation was even higher than the band intensity suggests. VHL mutational status is summarised by m=mutant; w=wild type. (**B**) In total, 30 *μ*g of tumour and normal lysates were analysed for levels of EGFR, phospho-RPS6 and total RPS6. The four cell line lysates were treated with Iressa and rapamycin (I/R) for 2 h or not (−). Densitometric analysis for the left panel was normalised to the tumour with the lowest EGFR signal. Densitometry for the right panel utilised a shorter exposure to remain within the linear response range. Even the longest exposures failed to detect EGFR in T-12. The asterisk denotes a background band. (**C**) Direct comparisons of phospho-Erk and phospho-Akt levels in a subset of tumours and cell lines on the same filter. (**D**) WT8^VHL-wt^ cultures starting at 70% confluency (time zero) were grown under standard culture conditions over 48 h and periodically sampled. Phospho-RPS6 levels were downregulated at 100% confluency.

Cell lines differ from primary tumours in terms of growth rates, altered microenvironment (nutrients, oxygen tension and extracellular matrix) and the absence of interacting stroma. To test for differences in phosphoprotein levels, we examined 12 clear-cell carcinomas and their corresponding uninvolved kidney (Figure 6). Table 1 (Methods) provides the clinical characteristics of these tumours including mutational analysis of VHL, which is summarised in Figure 6. Phospho-ERK1/2 was readily detectable and upregulated in nine of the 12 tumours (Figure 6A). Of note, most of these same tumour samples contained elevated levels of phospho-AKT. Total AKT, ERK and Coomassie staining are shown as controls. Compared to the cell lines, longer radiographic exposures were required to detect phospho-AKT. The difference in phospho-AKT was confirmed in side-by-side comparisons with selected tumours and cell lines (Figure 6C), while comparable levels of phospho-ERK were detected in both cell lines and tumours.

Levels of eIF4E were generally higher in the matched normal samples than in the tumours, with the exception of tumour #12 that had substantially increased eIF4E (compare lanes 23 and 24). Tumour 12 had sarcomatoid features (Table 1), indicating a more aggressive tumour and correspondingly poorer prognosis (Cheville *et al*, 2004). This tumour showed no detectable Erk phosphorylation despite abundant Erk protein and no detectable EGFR (Figure 6B). The biochemical characteristics of tumour 12 thus closely match the cell line SKRC-39 and verify that a subset of RCCs appear to be activated at this downstream translation regulatory point.

Most of the tumours expressed more EGFR protein than the corresponding normal tissue, yet EGFR levels were up to 30-fold higher in cell lines despite comparable levels of mRNA (Figure 6B, Tables 2 and 5). Among the tumours, only nos. 6 and 7 had EGFR protein levels that were as high as the cell lines and at least T-6 also expressed elevated EGFR message. In contrast, ErbB-3 mRNA was much higher in the primary tumours (and matched normal kidney) than in RCC cell lines, while ErbB-4 message was markedly reduced in tumours compared to normal (Table 5).

An additional major difference between tumours and cell lines involved phospho-RPS6, an indicator of mTOR activity. Phosphorylated RPS6 was undetectable in both tumours and normal kidney (Figure 6B), despite usually abundant levels of total RPS6 protein. Cell–cell contacts in solid tumours may result in alterations akin to contact inhibition in tissue culture. We therefore examined the effect of confluency on the phosphorylation state of RPS6 in WT8 cells (Figure 6D). While phospho-Akt and phospho-Erk were unaffected, phosphorylation of RPS6 on Ser235/236 was dramatically downregulated at 100% confluency. Thus, differences in RPS6 phosphorylation between tumours and cell lines may be a result of different growth rates or of cell–cell interaction.

## DISCUSSION

The results reported here demonstrate that combined inhibition of EGFR and mTOR provides a more effective growth blockade in RCC cell lines than either agent alone. Moreover, this effect was influenced by the mutational state of VHL. While we observed synergistic growth inhibition in both mutant and wt-VHL cell lines at high drug concentrations, synergy at Iressa doses near the IC_50_ for EGFR were limited to wt-VHL cells. Western blot analysis of phospho-proteins demonstrated that EGFR inhibition selectively blocked phosphorylation of ERK1/2 and RPS6 in cells with wt-VHL. The inhibition of RPS6 phosphorylation by Iressa, however, was not universal, which may relate to the presence of a PTEN mutation in 786-O and its derivatives (Kau *et al*, 2003). Mutant VHL lines showed little response to Iressa in terms of ERK and RPS6 phosphorylation and AKT phosphorylation was unaffected by either agent irrespective of VHL. These results indicate that wt-VHL cells are more sensitive to EGFR inhibition (or more dependent upon EGFR signalling for growth) than their mutant counterparts. Induction of HIF2*α* using cobalt treatments failed to prevent Iressa inhibition of Erk phosphorylation in WT8^VHL-wt^ cells, suggesting that this effect is independent of HIF.

Perera *et al* (2000) also noted that wt-VHL conveyed sensitivity to the EGFR blocking antibody, C225. However, changes in phospho-protein signalling were not described. Interestingly, we observed that the combination of low-dose Iressa and rapamycin was antagonistic in cells with mutant-VHL. This raises the possibility that certain drug targets might be regulated in an opposite manner depending on the state of VHL. Similar AKT-dependent results have been reported for single-agent rapamycin (Gera *et al*, 2004). However, since we did not detect changes in AKT phosphorylation, another target(s) that is VHL-dependent presumably would be responsible. Our studies suggest that there are at least three biochemical patterns in RCC cell lines: those responsive to EGFR inhibition in terms of ERK and RPS6 phosphorylation, those that are resistant and a third subset (likely resistant) with overexpression of eIF4E and downregulation of upstream signalling components. Of note, in cells where the eIF4E-binding protein 4E-BP1 is limiting, eIF4E overexpression also drives translation initiation in a rapamycin-resistant manner (Dilling *et al*, 2002).

During these investigations, we identified higher levels of EGFR protein in PRC3 cells (mutant VHL) compared to WT8. In agreement, EGFR phosphorylation following EGF stimulation was also prolonged. However, this appears not to be the explanation for differential Iressa sensitivity since pretreatment of cells with Iressa blocked EGFR phosphorylation equally. Also, EGFR protein levels were not affected by Iressa or rapamycin. We also noted that EGFR protein was generally higher in cell lines than primary tumours despite comparable mRNA levels. Whether this represents an *in vitro* selection phenomenon with preferential growth of these cells is unknown. Previous investigators have not reported suppression of EGFR protein after re-expression of wt-VHL (Knebelmann *et al*, 1998; Gunaratnam *et al*, 2003). However, in the report by Gunaratnam *et al*, (2003), adenovirus-mediated introduction of wt-VHL into 786-O cells visibly reduced EGFR levels (see their Figure 2A), although this difference was either discounted or not further clarified (Gunaratnam *et al*, 2003). In the report of Knebelmann *et al*, (1998), the Western blot appears too overexposed for optimal quantitation (Knebelmann *et al*, 1998). On the other hand, we have not detected reduced EGFR protein in MPR6, an independent wt-VHL derivative of 786-O. However, MPR6 expresses two anti-VHL reactive bands at considerably lower levels than the single band detected (with either anti-HA or anti-VHL antibodies) in WT8. Thus, we cannot conclude that the changes in EGFR are a consequence of VHL. This difference could, for example, be explained by an acquired clonal variation affecting some aspect of EGFR protein processing or degradation. In any case, since EGFR mRNA levels in 786-O, PRC3 and WT8 were essentially identical, and since we were unable to detect consistent differences in EGFR ribosome association between PRC3 and WT8, we suspect that the difference involves EGFR degradation, which would be consistent with the observed differences in the duration of receptor phosphorylation. This would also be consistent with the absence of EGFR mRNA among a set of messages whose translation initiation was affected by VHL mutation (Galban *et al*, 2003).

EGFR overexpression in RCC has previously been well documented (Gomella *et al*, 1989; Uhlman *et al*, 1995). The finding that TGF*α* is constitutively expressed as a consequence of VHL mutations (de Paulsen *et al*, 2001), and that TGF*α* is a mitogen for renal epithelial cells, considerably strengthened the hypothesis that EGFR signalling is important in RCC development. However, in contrast to lung cancer, activating mutations in exons 19 and 21 of EGFR were not detected in 16 kidney tumours (Lynch *et al*, 2004) and, clinically, tumour regressions following EGFR inhibition have been minimal (Amato, 2005). If EGFR signalling is deregulated in RCC, then why are clinical responses so limited? Possible explanations include involvement of additional ErbB family members not affected by selective EGFR inhibitors, activation of other growth factor pathways such that EGFR blockade is insufficient to induce tumour regression, and changes in apoptotic components that prevent cell death in response to EGFR blockade.

Regarding other ErbB family members, in cell lines we found that EGFR was by far the predominant component among mRNAs and the only ErbB protein detectable by Western analysis. For ErbB-2, previous reports have been inconsistent. Stumm *et al* (1996) reported that p185erbB-2 was overexpressed in RCC while Freeman *et al*, (1989) found ErbB-2 to be reduced (Freeman *et al*, 1989; Stumm *et al*, 1996). We found ErbB-2 mRNA was expressed at low levels in both cell lines and primary tumours. For ErbB-3 and ErbB-4, Thomasson *et al* (2004) reported that both receptors were downregulated. Our results are in agreement for ErbB-4, although ErbB-3 was discordant, being downregulated in cell lines but maintained at substantial levels in primary tumours. Potentially, this is an important difference although the biological consequences are unclear. Although ErbB-3 lacks kinase activity (Burgess *et al*, 2003), it couples efficiently to phosphatidylinositol 3-kinase (Fedi *et al*, 1994; Burgess *et al*, 2003), has distinct preferred ligands (i.e. NDF, HB-EGF and BTC)(Beerli and Hynes, 1996) and is endocytosed less readily than EGFR (Baulida *et al*, 1996). Interestingly, in mice one of these ligands, NDF, is most highly expressed in the kidney and during development correlates with urogenital organ formation (Castagnino *et al*, 2000). At least in keratinocytes, NDF is induced by hepatocyte growth factor (HGF) (Castagnino *et al*, 2000). If this occurred in the kidney, then RCCs with VHL mutations, which are known to upregulate MET and be hyper-responsive to HGF (Oh *et al*, 2002; Koochekpour *et al*, 1999), might behave differently in terms of ErbB signalling and EGFR inhibition than cell lines.

Regarding other growth factor pathways that might render EGFR blockade insufficient, we note that phospho-AKT was not affected by short-term Iressa, rapamycin or the combination. Similarly, phospho-ERK1/2 showed no response to Iressa in mutant VHL cell lines. These results strongly suggest that other growth factor signalling pathways are activated. The inability of Iressa and rapamycin to affect AKT led us to ask whether blocking AKT activation with the PI3K, LY294002, would alter protein translation initiation of MYC. We chose MYC because of its overexpression in RCC (Yao *et al*, 1988) and because it contains an internal ribosome entry site that may allow initiation in an eIF4E-independent manner. MYC mRNA polysome loading was not affected by Iressa or rapamycin while the control RPS19 was substantially downregulated. In contrast, a strong reduction in MYC polysome loading was achieved by 2 h PI3K inhibition.

Lastly, how relevant are the phospho-protein analyses to primary tumours? Levels of phospho-ERK1/2 were similar between cell lines and primary tumours and these same tumours also had elevated phospho-AKT, although less so than in cell lines. Thus, phospho-ERK and AKT may be useful biomarkers in RCC. While most tumours overexpressed EGFR compared to matched normal controls, only two of 12 contained EGFR levels comparable to those observed in RCC cell lines. Another striking difference was the absence of RPS6 phosphorylation in any of the tumour/normal samples despite easily detectable total RPS6 protein. RPS6 is phosphorylated by p70S6K, which in turn is activated by mTOR (Hay and Sonenberg, 2004). Similar results were suggested by Kenerson *et al* (2002) who found increased phospho-RPS6 in RCCs derived from patients with tuberous sclerosis but not in sporadic RCCs (Kenerson *et al*, 2002). Other studies of non-RCC primary tumours have frequently identified mTOR activation. A broad study of phosphorylated S6K1 in eight common tumour types found nearly all with some reactivity while intense staining (3+) was present in up to 60% of colon adenocarcinomas and nearly 50% of lymphomas, ovarian, melanoma, lung and brain tumours (Xu *et al*, 2004).

Our results suggest that mTOR may be relatively inactive in primary RCCs. However, there are two important caveats. The first is that mTOR activity could be confined to areas of growth or invasion that would have been missed by our examination of bulk tumour protein. This interpretation is strengthened by our observation that confluency dramatically affected phospho-RPS6 levels in WT8 cells. Alternatively, mTOR may be activated in only a subset of RCCs in agreement with the observed 7% response rate to CCI-779 (Atkins *et al*, 2004).

One cell line (SKRC-39) expressed high levels of eIF4E in the absence of upstream signalling, and one tumour (#12) showed a very similar biochemical pattern, with high eIF4E but no detectable EGFR or phospho-Erk. Interestingly, this tumour had sarcomatoid differentiation, an indicator of more aggressive behaviour and poor prognosis (Cheville *et al*, 2004). Thus, eIF4E overexpression appears to define a subset of RCCs that are likely to be resistant to rapamycin and/or Iressa, consistent with the aggressive nature of sarcomatoid RCCs.

In summary, our results provide rationale for the testing of combined EGFR and mTOR inhibitors, at least in the subset of RCC patients with wt-VHL. It will be important to determine whether mTOR activation is heterogeneous or activated in only certain subsets of tumours and whether *in vivo* phospho-AKT is affected by these treatments.

## Figures and Tables

**Table 1:** Clinical characteristics and von Hippel–Lindau (VHL) status of 12 RCC tumours

**Tumour**	**Dx**	**Other features**	**T stage**	**Furhman grade**	**% tumour**	**VHL status**	**Specific alteration**
1	Clear cell		1b	2	100	mut	N78S
2	Clear cell		2	3	100	mut	V155E or L
3	Clear cell		1b	2	100	mut	V155E or L
4	Clear cell		3a	2	100	WT	
5	Clear cell		1b	2	100	mut	L158Q
6	Clear cell		3a	3	100	mut	L158P
7	Clear cell		3a	4	90	mut	V155E or L
8	Clear cell		3a	4	60	WT	
9	Clear cell		1a	2	20	WT	
10	Clear cell	Cystic	1a	2	20	mut	W88C
11	Clear cell	Cystic	1b	2	30	mut	L128H
12	Clear cell	Sarcomatoid	3b	4	100	WT	

**Table 2:** Expression^a^ of ErbB family genes and von Hippel–Lindau (VHL) mutant status in renal cell carcinoma (RCC) cell lines

**Sample**	**EGFR**	**ErbB-2**	**ErbB-3**	**ErbB-4**	**VHL mutation**	**VHL exp.**
PV10	0.3	0	0.003	0	ND^b^	
KRCY	0.6	0	0.028	0	Wt	0.3
SKRC39	0.8	0.018	0.009	0.030	Wt	1.2
KV6	1.8	0	0.0004	0	Wt	0.01
A498	2.0	0	0.002	0.002	V142/D143-f.s.^c^	
ACHN	2.2	0.002	0.142	0	Wt^d^	0.2
SKRC48	4.3	0	0.006	0.001	L135-f.s.	
SKRC12	4.5	0	0.009	0	N121Y	
CAKI2	4.7	0.002	0.309	0	ND	
A704	5.8	0.001	0.303	0	ND	
SKRC17	6.3	0.003	0.002	0.001	S68->stop	
SKRC02	8.0	0.001	0.001	0	S68P	
PRC3	9.3	0.002	0.002	0.010	ΔG104-f.s.	
SKRC45	15.1	0.005	0.059	0	R82P	

a Expressed as % of GAPDH.

b Not determined.

c f.s.=frameshift.

d Differs from lit report.

**Table 3:** Expression^a^ of ErbB family genes in 786-O cell lines

**Line**	**EGFR**	**ErbB-2**	**ErbB-3**	**ErbB-4**	**VEGF**
786-O	2.0	0.0009	0.0003	0.0001	1.1
PRC3	1.6	0.0005	0.0002	0.0005	0.8
WT8	2.3	0.0005	0.0005	0.0003	0.3

a Expressed as % of *β*-actin.

EGFR=epidermal growth factor receptor; VEGF=vascular endothelial growth factor.

**Table 4:** mRNA^a^ loading on polysomes in WT8 cells

**Gene**	**Treatment**	**1–2 rib**	**3–4 rib**	**Polyrib**
MYC	DMSO	0.20	0.16	0.08
	Iressa	0.11	0.09	0.09
	Rapamycin	0.40	0.23	0.20
	Iressa+Rapamycin	0.36	0.47	0.14
				
MYC	DMSO	0.31	0.42	0.24
	LY294002	0.05	0.16	0.15
	Ly+Rap	0.05	0.07	0.09
	Ly+Rap+Iressa	0.03	0.05	0.09
				
RPS19	DMSO	319.3	117.7	10.6
	Iressa	149.0	33.9	4.5
	Rapamycin	199.3	32.9	3.3
	Iressa+Rapamycin	101.4	52.5	2.6

a Expressed as % *β*-actin.

DMSO=dimethyl sulphoxide.

**Table 5:** Expression^a^ of mRNA for ErbB Genes in renal cell carcinoma (RCC) tumours

**Sample**	**EGFR**	**ErbB-2**	**ErbB-3**	**ErbB-4**
1-N	5.5	0.04	3.2	14.8
2-N	1.7	0.01	3.0	2.4
3-N	1.8	0.01	3.0	1.3
4-N	1.3	0.01	1.8	5.7
5-N	1.7	0.01	2.8	1.6
6-N	5.5	0.03	1.7	1.6
1-T	2.7	0.002	1.0	0.0003
2-T	1.9	0.002	2.2	0.001
3-T	1.9	0.001	1.9	0.005
4-T	3.1	0.002	0.6	0.017
5-T	2.5	0.001	0.4	0.020
6-T	13.2	0.004	2.8	0.035

a Expressed as % of GAPDH. EGFR=epidermal growth factor receptor; N=normal adjacent kidney; T=tumour.
